# Cardiometabolic index: A new predictor for metabolic associated fatty liver disease in Chinese adults

**DOI:** 10.3389/fendo.2022.1004855

**Published:** 2022-09-16

**Authors:** Shaojie Duan, Deshuang Yang, Hui Xia, Zhiying Ren, Jialiang Chen, Shukun Yao

**Affiliations:** ^1^ Graduate School, Beijing University of Chinese Medicine, Beijing, China; ^2^ Department of Gastroenterology, China-Japan Friendship Hospital, Beijing, China; ^3^ Department of Integrative Cardiology, China-Japan Friendship Hospital, Beijing, China; ^4^ Center of Integrative Medicine, Beijing Ditan Hospital, Capital Medical University, Beijing, China

**Keywords:** cardiometabolic index, metabolic associated fatty liver disease, receiver operating characteristic curve, predictor, diagnosis

## Abstract

**Objective:**

Cardiometabolic index (CMI) is a well promising indicator for predicting obesity-related diseases, but its predictive value for metabolic associated fatty liver disease (MAFLD) is unclear. This study aimed to investigate the relationship between CMI and MAFLD and to evaluate the predictive value of CMI for MAFLD.

**Methods:**

A total of 943 subjects were enrolled in this cross-sectional study. CMI was calculated by multiplying the ratio of triglycerides and high-density lipoprotein cholesterol (TG/HDL-C) by waist-to-height ratio (WHtR). Multivariate logistic regression analysis was used to systematically evaluate the relationship between CMI and MAFLD. Receiver operating characteristic (ROC) curves were used to assess the predictive power of CMI for MAFLD and to determine the optimal cutoff value. The diagnostic performance of high CMI for MAFLD was validated in 131 subjects with magnetic resonance imaging diagnosis.

**Results:**

Subjects with higher CMI exhibited a significantly increased risk of MAFLD. The odds ratio for a 1-standard-deviation increase in CMI was 3.180 (2.102-4.809) after adjusting for various confounding factors. Further subgroup analysis showed that there were significant additive interactions between CMI and MAFLD risk in gender, age, and BMI (*P* for interaction < 0.05), and the area under the ROC curve(AUC) of CMI for predicting MAFLD were significantly higher in female, young, and nonobese subgroups than that in male, middle-aged and elderly, and obese subgroups (all *P* < 0.05). Moreover, among nonobese subjects, the AUC of CMI was significantly higher than that of waist circumference, BMI, TG/HDL-C, and TG (all *P* < 0.05). The best cutoff values of CMI to diagnose MAFLD in males and females were 0.6085 and 0.4319, respectively, and the accuracy, sensitivity, and specificity of high CMI for diagnosing MAFLD in the validation set were 85.5%, 87.5%, and 80%, respectively.

**Conclusions:**

CMI was strongly and positively associated with the risk of MAFLD and can be a reference predictor for MAFLD. High CMI had excellent diagnostic performance for MALFD, which can enable important clinical value for early identification and screening of MAFLD.

## Introduction

Metabolic associated fatty liver disease (MAFLD) is the leading cause of chronic hepatic disease (1). MAFLD affects a quarter of the world’s adult population and places an enormous burden on individuals, families, and healthcare systems (2, 3). Further deterioration of MAFLD can not only lead to hepatitis, liver fibrosis, cirrhosis, liver cancer (4–8), but also enhance the risk of other metabolic dysfunctions, such as insulin resistance, diabetes, dyslipidemia, chronic kidney disease, cardiovascular and cerebrovascular diseases (9–12). Therefore, early diagnosis and intervention are very necessary to reduce the burden of MAFLD.

Liver biopsy is well known as the “gold standard” for MAFLD, but it is limited in clinical application due to invasive, difficult operation, high price, and poor patient compliance. Ultrasound is the most commonly used clinical diagnostic method, but the diagnostic sensitivity of mild steatosis is poor and is often affected by the level of equipment and operators. Computed tomography (CT) has good diagnostic sensitivity, but its application is limited by radiation. Magnetic resonance imaging (MRI), due to its noninvasive nature, high precision and high reproducibility, has become the method of choice for quantification noninvasive liver fat in clinical and research, but it has the limitation of lower specificity and higher cost (3, 13, 14). Therefore, it is of great significance to find a low-cost and simple auxiliary diagnostic method for the early identification of MAFLD.

One of the hallmarks of MAFLD is hepatic steatosis. It is characterized by excessive accumulation of triglyceride (TG) and cholesterol in lipid droplets (LDs) in hepatocytes caused by visceral fat accumulation (15). However, the main risk factors for visceral fat accumulation and hepatic steatosis are central obesity and insulin resistance (16). Therefore, obesity-related body fat indicators are of great value for MAFLD. Studies found that MAFLD was closely related to body mass index (BMI), waist circumference (WC), waist-to-height ratio (WHtR), aminotransferase, fatty acid, serum levels of triglycerides (TG), total cholesterol (TC), high-density lipoprotein cholesterol (HDL-C), and the ratio of TG and HDL-C (TG/HDL-C) (17–20). Among them, the TG/HDL-C was confirmed as the predictor of nonalcoholic fatty liver disease, and it can identify insulin resistance, abdominal obesity, metabolic disorders, and cardiometabolic risk (11–25). The WHtR was identified as an optimal anthropometric indicator for MAFLD in the Western Chinese male population (17).

Recently, the cardiometabolic index (CMI), calculated by multiplying TG/HDL-C by WHtR, was a new index to evaluate the distribution and dysfunction of visceral adipose tissue (22). Previous studies found that CMI was closely associated with cardiovascular disease, kidney disease, and adverse metabolic, and it might be a well promising indicator for predicting metabolism-related diseases (26–29). However, the predictive value of CMI on MAFLD has not been researched.

Therefore, this study aimed to systematically investigate the relationship between CMI and MAFLD, evaluate the predictive value of CMI for MAFLD and determine the optimal cutoff value of CMI for diagnosing MAFLD in different genders. Then, we will further verify the diagnostic performance of high CMI for MAFLD by MRI diagnosis, aiming to provide new ideas for the early prevention and screening of MAFLD.

## Materials and methods

### Study design and participants

**Figure 1:**
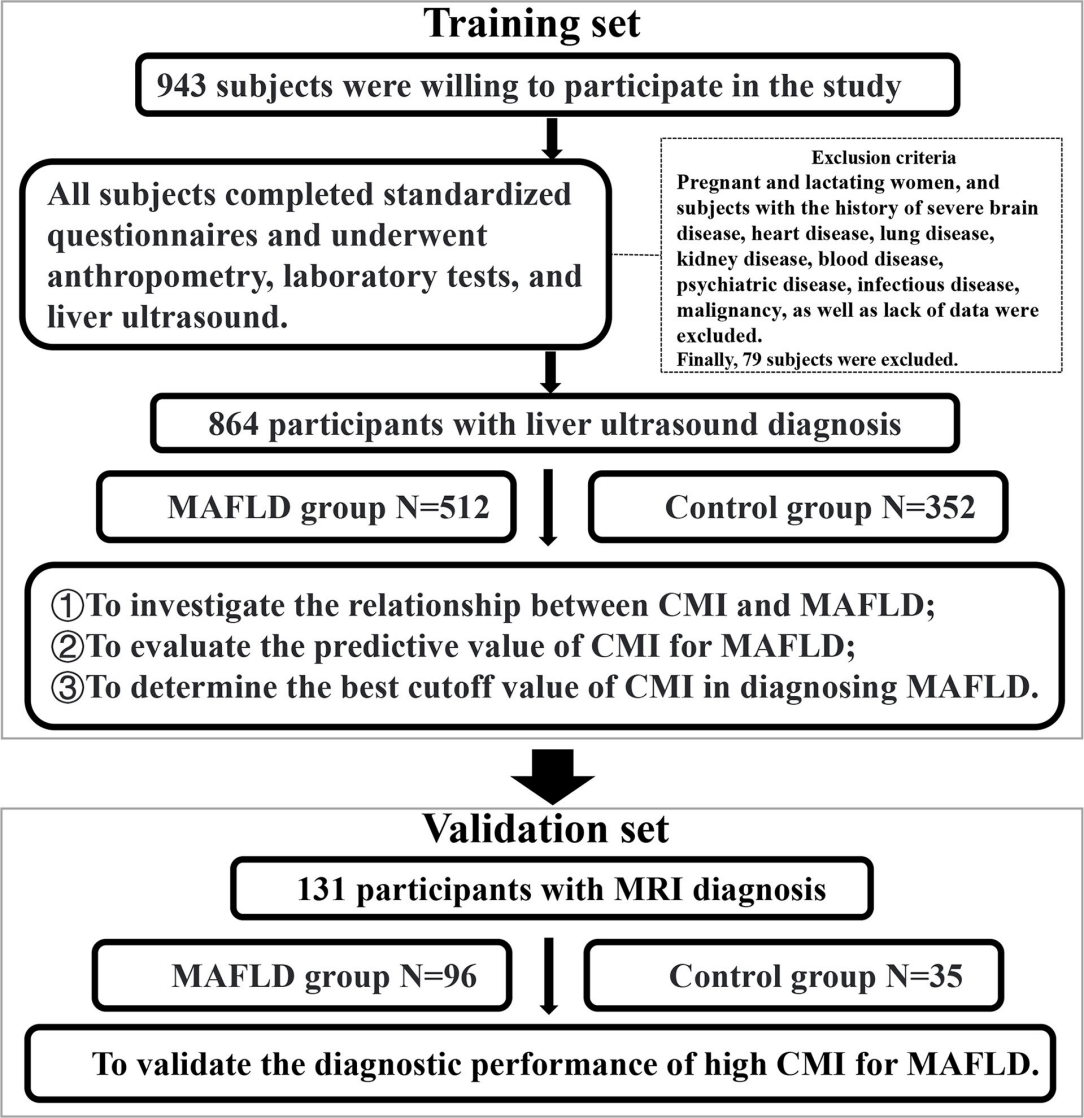
Flow chart of the study subjects.

This cross-sectional study was conducted at the Health Examination Center of China-Japan Friendship Hospital in Beijing, China. The study lasted about three years from September 2018 to October 2021. A total of 943 individuals who underwent physical examinations were willing to participate in the study. They filled out standardized questionnaires under the guidance of physicians. And they underwent anthropometry, laboratory tests and liver ultrasound examination. Pregnant and lactating women, and subjects with the history of severe brain disease, heart disease, lung disease, kidney disease, blood disease, psychiatric disease, infectious disease, malignancy, as well as lack of data were excluded. Finally, 864 participants with liver ultrasound diagnosis were recruited, including 624 males and 240 females, aged 20 to 78 years (**Figure 1**).

This study has been approved by the Clinical Research Ethics Committee of China-Japan Friendship Hospital (2018-110-K79-1). All subjects voluntarily agreed to participate in this study and submitted informed consent forms.

### Data collection and definition

The physical examinations were performed in the morning with a fasting state. Anthropometric indicators were measured by professionally trained physicians. Height, weight, and waist circumference (WC) were measured while subjects were naturally standing without shoes and heavy clothing. After 10 minutes of rest, the blood pressure was measured by the upper arm electronic sphygmomanometer. Peripheral blood was drawn from the cubital vein into a tube containing EDTA and sent to the laboratory of China-Japan Friendship Hospital for testing within 2 hours. The obtained peripheral venous blood will be used to detect the following indicators through Chemistry Analyzer(Instrument name and model: BECKMAN COULTER Chemistry Analyzer AU5800, Beckman Kurt Co., Ltd, US), including alanine aminotransferase (ALT), aspartate aminotransferase (AST), total cholesterol (TC), triglyceride (TG), high-density lipoprotein cholesterol (HDL-C), low-density lipoprotein cholesterol (LDL-C), fasting blood glucose (FBG), serum uric acid (SUA).

Body mass index (BMI) was calculated as the weight (in kilograms) divided by the square of the height (in meters). WC was the circumference at the level of the flat navel. CMI was calculated as the product of WHtR and TG/HDL-c.

### Detection and definition of MAFLD

In the training set, fatty liver (hepatic steatosis) was defined by professional sonographers through liver ultrasound examination, which required at least two of the following three manifestations: diffusely increased echogenicity of the liver relative to the kidney or spleen, ultrasound beam attenuation, or poor visualization of intrahepatic structures. The diagnostic criteria of MAFLD referred to the consensus of international experts in 2020: In addition to the diagnosis of hepatic steatosis (1), also need to meet one of the following three, namely, overweight/obesity, type 2 diabetes mellitus, and metabolic dysregulation that included at least two of the following metabolic risk criteria: 1) Waist circumference ≥102/88 cm in Caucasian men and women or ≥90/80 cm in Asian men and women; 2) Blood pressure ≥130/85 mmHg or specific drug treatment; 3) Plasma triglycerides ≥1.70 mmol/L or specific drug treatment; 4) Plasma HDL-cholesterol <1.0 mmol/L for men and <1.3 mmol/L for women or specific drug treatment; 5) Prediabetes (i.e., fasting glucose levels 5.6 to 6.9 mmol/L, or 2-hour post load glucose levels 7.8 to 11.0 mmol or HbA1c 5.7% to 6.4%; 6) Homeostasis model assessment (HOMA)-insulin resistance score ≥2.5; 7) Plasma high-sensitivity C-reactive protein (hs-CRP) level >2 mg/L (30, 31).

In the validation set, the proton density fat fraction (PDFF) based on magnetic resonance imaging (MRI) was used to diagnose fatty liver. MAFLD was defined when PDFF ≥ 5% (32).

### Statistical analysis

First, the baseline characteristics of the MAFLD and non-MAFLD groups were compared. The quantitative data of normal distribution between groups were expressed as mean ± standard deviation. The independent samples *t* test was used to compare normally distributed quantitative data between groups. The quantitative data of non-normal distribution were represented by median and quartile. The Mann–Whitney *U* test was used to compare non-normally distributed quantitative data between groups. The categorical data were expressed in numbers and percentages. The chi-squared test was used to compare categorical data between groups.

All statistical tests were two-tailed and were considered significant for P less than 0.05 (P <0.05). Statistical analyses were performed using Statistical Package for the Sciences (SPSS, version 25.0) and MedCalc statistical software (version 19.6.4).

## Results

### Characteristics of participants

The demographics, anthropometrics, and laboratory test characteristics of 864 subjects were presented in **Table 1**. The prevalence of male and young patients (age<45 years old), the percentage of smoking history and drinking history in MAFLD group were significantly higher than those in the control group (all *P* < 0.05). Participants with MAFLD had dramatically higher levels of CMI, WC, BMI, SBP, DBP, ALT, AST, TC, TG, LDL-C, FBG, and SUA and significantly lower HDL-C levels (all *P* < 0.05).

**Table 1:** Baseline characteristics of the MAFLD group and control group.

Variable	Total (n=864)	Control (n=352)	MAFLD (n=512)	*P-*value^a^
Gender [n (%)]				< 0.001
Male	624 (72.7)	230 (65.3)	394 (77.0)	
Female	240 (27.8)	122 (34.7)	118 (23.0)	
Age,years [M (P25-P75)]	37.0 (32.0-47.0)	35.0 (30.0-43.0)	39.0 (33.0-48.0)	< 0.001
Age≥45 years [n (%)]	265 (69.3)	79(22.4)	186 (36.3)	< 0.001
Age<45 years [n (%)]	599 (30.7)	273(77.6)	326 (63.7)	
Smoking history [n (%)]	255 (29.5)	81 (23.0)	174 (34.0)	< 0.001
Drinking history [n (%)]	221 (25.6)	76 (21.6)	145 (28.3)	0.027
WC, cm [M (P25-P75)]	93.0 (86.0-99.0)	86.0 (79.5-93.0)	96.0 (91.0-102.0)	< 0.001
BMI, kg/m^2^ [M (P25-P75)]	26.49 (24.26-28.73)	24.45 (22.15-26.56)	27.74 (25.85-29.74)	< 0.001
SBP, mmHg [M (P25-P75)]	130.0 (120.0-139.0)	125.0 (116.0-135.0)	133.0 (123.0-142.0)	< 0.001
DBP, mmHg [M (P25-P75)]	81.0 (72.0-88.0)	77.5 (70.0-85.0)	82.0 (75.0-90.0)	< 0.001
ALT, U/L [M (P25-P75)]	28.0 (19.0-42.0)	21.0 (15.0-30.0)	34.0 (24.0-54.0)	< 0.001
AST, U/L [M (P25- P75)]	21.0 (18.0-26.0)	19.0 (17.0-23.0)	23.0 (19.0-29.0)	< 0.001
TC, mmol/L [M (P25-P75)]	4.61 (4.11-5.25)	4.44 (1.04-5.00)	4.77 (4.17-5.41)	< 0.001
TG, mmol/L [M (P25-P75)]	1.47 (0.98-2.19)	1.06 (0.74-1.48)	1.84 (1.28-2.59)	< 0.001
HDL-C, mmol/L [M (P25-P75)]	1.23 (1.06-1.42)	1.34 (1.15-1.52)	1.14 (1.01-1.33)	< 0.001
LDL-C, mmol/L [M (P25-P75)]	2.72 (2.30-3.26)	2.59 (2.15-3.08)	2.84 (2.39-3.37)	< 0.001
FBG, mmol/L [M (P25-P75)]	5.29 (4.98-5.72)	5.15 (4.90-5.43)	5.40 (5.07-5.88)	< 0.001
SUA, μmol/L [M (P25-P75)]	357.5 (296.3-418.8)	325.5 (262.3-394.0)	375.5 (314.0-434.0)	< 0.001
CMI [M (P25-P75)]	0.68 (0.39-1.13)	0.39 (0.25-0.64)	0.88 (0.61-1.40)	< 0.001

Data are presented as median with the interquartile range [M (P25-P75)], or frequency (percentage) [n (%)]. ^a^ Comparison of the differences between the two groups calculated by Mann-Whitney U test or chi-square test. MAFLD, metabolic associated fatty liver disease; WC, waist circumference; BMI, body mass index; SBP, systolic blood pressure; DBP, diastolic blood pressure; ALT, alanine aminotransferase; AST, aspartate aminotransferase; TC, total cholesterol; TG, triglyceride; HDL-C, high-density lipoprotein cholesterol; LDL-C, low-density lipoprotein cholesterol; FBG, fasting blood glucose; SUA, serum uric acid; CMI, cardiometabolic index.

### Multivariate logistic regression analysis of CMI on the risk of MAFLD

Multivariate logistic regression analyses were used to explore the relationship between CMI and MAFLD risk, and the results were shown in **Table 2**. CMI had a strong association with the risk of MAFLD, and the OR for a 1-standard-deviation (1-SD) increase in CMI was 9.54 (6.357-14.318) without adjustment (Model 1). After adjusting for gender and age, the OR for a 1-SD increase in CMI was 9.139 (6.102-13.688) (Model 2). After further adjusting for smoking history, drinking history, WC, and BMI, the degree of this association changed but was still strong, there was a 3.714-fold (2.463-5.601) higher risk for MAFLD with a 1-SD increase in CMI (Model 3). Further adjusting for SBP, DBP, ALT, AST, TC, TG, HDL-C, LDL-C, FBG, and SUA attenuated the association but not too much, there was still a 3.180-fold (2.102-4.809) higher risk for MAFLD with a 1-SD increase in CMI (Model 4).

**Table 2:** Multivariate logistic regression of CMI for MAFLD.

Variable	β	SE	Wald χ^2^	*P*-value	OR (95%CI)
Model1					
CMI level (per SD change)	2.256	0.207	118.543	< 0.001	9.54 (6.357-14.318)
Quartiles of CMI					
C1 (≤ 0.3867)	——	——	——	——	
C2 (0.3867~0.6777)	1.512	0.217	48.457	< 0.001	4.535 (2.963-6.940)
C3 (0.6777~1.1268)	2.537	0.233	118.771	< 0.001	12.647 (8.013-19.961)
C4 (>1.1268)	3.227	0.261	152.319	< 0.001	25.207 (15.099-42.081)
Model2					
CMI level (per SD change)	2.213	0.206	115.215	< 0.001	9.139 (6.102-13.688)
Quartiles of CMI					
C1 (≤ 0.3867)	——	——	——	——	1 (Ref)
C2 (0.3867~0.6777)	1.455	0.219	44.137	< 0.001	4.285 (2.789-6.583)
C3 (0.6777~1.1268)	2.457	0.235	109.699	< 0.001	11.675 (7.371-18.492)
C4 (>1.1268)	3.208	0.263	149.202	< 0.001	24.732 (14.781-41.383)
Model3					
CMI level (per SD change)	1.312	0.21	39.201	< 0.001	3.714 (2.463-5.601)
Quartiles of CMI					
C1 (≤ 0.3867)	——	——	——	——	1 (Ref)
C2 (0.3867~0.6777)	0.91	0.244	13.911	< 0.001	2.484 (1.54-4.006)
C3 (0.6777~1.1268)	1.618	0.266	36.898	< 0.001	5.042 (2.991-8.497))
C4 (>1.1268)	2.251	0.299	56.767	< 0.001	9.498 (5.288-17.058)
Model4					
CMI level (per SD change)	1.157	0.211	30.031	< 0.001	3.1802.102-4.809)
Quartiles of CMI					
C1 (≤ 0.3867)	——	——	——	——	1 (Ref)
C2 (0.3867~0.6777)	0.865	0.252	11.801	0.001	2.375 (1.45, 3.891)
C3 (0.6777~1.1268)	1.54	0.274	31.650	< 0.001	4.665 (2.728, 7.977)
C4 (>1.1268)	2.091	0.307	46.497	< 0.001	8.093 (4.437, 14.762)

Model 1: Unadjusted. Model 2: Adjusted for age and gender. Model 3: Adjusted for age, gender, smoking history, drinking history, waist circumference, and body mass index. Model 4: Adjusted for age, gender, smoking history, drinking history, waist circumference, body mass index, systolic blood pressure, diastolic blood pressure, alanine aminotransferase, aspartate aminotransferase, total cholesterol, triglyceride, high-density lipoprotein cholesterol, low-density lipoprotein cholesterol, fasting blood glucose, and serum uric acid.

After dividing CMI into quartiles, the risk of MAFLD still increased significantly with increasing CMI quartiles. When comparing the top quartiles with the bottom categories, the risk of MAFLD increased 25.207-fold to 8.093-fold from Model 1 to Model 4. The *P* values for the linear trend were less than 0.01, indicating d that the linear trends from the lowest to the highest quartiles were eminent.

### Effect of CMI on the risk of MAFLD stratified by subgroups

To further investigate the impact of other risk factors on the correlation between CMI and MAFLD, subgroup analyses were carried out according to gender, age, BMI, history of smoking and drinking. **Table 3** summarized the results of the subgroup analysis and the interaction results.

**Table 3:** Effect of magnitude of CMI on MAFLD risk stratified by subgroups.

Characteristics	No. of participation	OR (95%CI)	*P-value*	*P* for interaction
Age				0.004
≥45 years	265	1.540 (0.617, 3.846)	0.355	
<45 years	599	2.366 (1.253, 4.469)	0.008	
Gender				0.003
Males	624	1.605 (0.944, 2.728)	0.080	
Females	240	32.284 (4.061, 256.674)	0.001	
Smoking history				0.919
Yes	255	0.873 (0.412, 1.849)	0.723	
No	609	5.024 (2.327, 10.849)	< 0.001	
Drinking history				0.309
Yes	221	1.328 (0.546, 3.228)	0.531	
No	643	3.315 (1.685, 6.522)	0.001	
BMI				0.006
≥28 kg/m^2^	282	0.629 (0.313, 1.264)	0.193	
<28 kg/m^2^	582	4.205 (2.105, 8.400)	< 0.001	

Adjusted for age, sex, smoking history, drinking history, waist circumference, body mass index, systolic blood pressure, diastolic blood pressure, alanine aminotransferase, aspartate aminotransferase, total cholesterol, triglyceride, high-density lipoprotein cholesterol, low-density lipoprotein cholesterol, fasting blood glucose, and serum uric acid.

After adjusting for age, sex, smoking history, drinking history, WC, BMI, SBP, DBP, ALT, AST, TC, HDL-C, LDL-C, FBG, and SUA, there were still significant additive interactions between CMI and MAFLD risk in gender, age, and BMI subgroups (*P* for interaction < 0.05). Stronger correlations were found in the participants with an age < 45 years old, BMI< 28 kg/m^2^, or females. However, significant interactions were not found in participants with the history of smoking and drinking.

### Predictive ability of CMI for MAFLD in different subgroups

**Figure 2:**
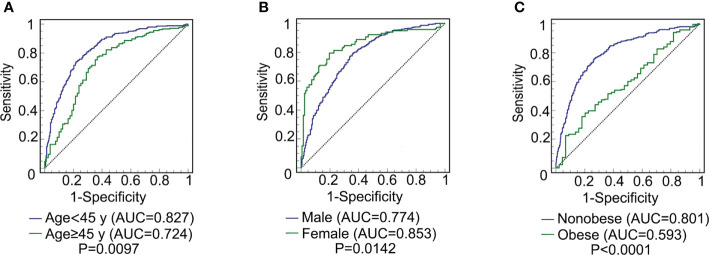
ROC curve comparison of CMI prediction of MAFLD among different subgroups. **(A)** comparison between young (age < 45) and middle-age and elderly (age ≥ 45 y) subjects (*P* = 0.0097); **(B)** comparison between male and female subjects (*P* = 0.0142); **(C)** comparison between non-obese and obese subjects (*P* < 0.0001). CMI, cardiometabolic index; MAFLD, metabolic-associated fatty liver disease; ROC, receiver operating characteristic; AUC, area under the ROC.

The ROC curve of CMI for predicting MAFLD in different sex, age, and weight subgroups was plotted. The DeLong test was used to compare the area under the ROC curve (AUC) between the subgroups. As shown in **Figure 2**, the AUC of CMI for MAFLD in the young subjects was significantly higher than that in middle-aged and elderly subjects [0.827(0.794-0.856) vs 0.724(0.666-0.777), *P* = 0.0097]. The AUC of CMI for MAFLD in females was significantly higher than that in males [0.853(0.801-0.895) vs 0.774(0.739-0.806), *P* = 0.0142]. The AUC of CMI for MAFLD in nonobese people was significantly higher than that in obese people [0.801(0.767-0.833) vs 0.593(0.533-0.651), *P < *0.0001].

In addition, the predictive ability of CMI, WC, BMI, TG/HDL-C, and TG for MAFLD in different subgroups were compared, and the results were exhibited in **Supplementary Table 1**. The AUC of CMI for predicting MAFLD was significantly higher than TG/HDL-C and TG in total subjects (all *P* < 0.05). Moreover, among nonobese subjects, the AUC of CMI was significantly higher than that of WC, BMI, TG/HDL-C, and TG (all *P* < 0.05).

### Validation of high CMI in predicting MAFLD

From the above results, the best cutoff values of CMI for predicting MAFLD in males and females were 0.6085 and 0.4319, respectively, so we defined high CMI when CMI > 0.6085 in males or CMI > 0.4319 in females. To further validate the predictive power of high CMI for MAFLD, we selected 131 subjects with MRI diagnosis for validation. Among them, 96 subjects were diagnosed with MAFLD by MRI and 35 were not. High CMI predicted MAFLD positive in 91 cases and negative in 40 cases. As calculated from **Table 4**, the diagnostic accuracy of high CMI for MAFLD was 85.5%, the sensitivity and specificity were 87.5% and 80%, respectively, and the positive and negative likelihood ratios were 4.375, 0.156, respectively.

**Table 4:** Crosstab of diagnostic tests for high CMI diagnosis of MAFLD.

Predictive MAFLD	Actual MAFLD	
	positive	negative
positive	84	7
negative	12	28

MAFLD, metabolic associated fatty liver disease.

Accuracy = 85.5%, sensitivity = 87.5%, specificity = 80%, positive likelihood ratio = 4.375, negative likelihood ratios = 0.156.

In addition, we also found that the CMI was positively associated with PDFF in the validation set (r=0.573, *P < *0.001). With the level of CMI increased, the PDFF level also increased significantly, which indicated that CMI had a positive correlation with liver fat content.

### The correlation between CMI and MAFLD related indicators

Spearman correlation test was carried out to analyze the correlation between CMI and MAFLD related indicators, and the results were shown in **Supplementary Table 2**. CMI had a strong positive correlation with WC, BMI, TG (r > 0.5, P < 0.01), a strong negative correlation with H-DLC (r < -0.5, P < 0.01), a moderate positive correlation with ALT, AST, SUA, FBG, SBP, SDP (r > 0.3, P < 0.01), and a weak correlation with TC, L-DLC (r > 0.1, P < 0.01).

## Discussion

This study systematically analyzed the relationship between CMI and MAFLD and evaluated the predictive value of CMI for MAFLD. We found that subjects with higher CMI had a higher risk of MAFLD through multivariate regression analysis. Compared with male, middle-aged and elderly, and obese subjects, there were significantly higher predictive ability of CMI for MAFLD in female, young and non-obese subjects. Further, we also determined the best cutoff values of CMI for diagnosing MAFLD in both genders, and 131 subjects with MRI diagnosis were selected to validate the diagnostic performance of high CMI for MAFLD, with the accuracy, sensitivity, and specificity being 85.5%, 87.5%, and 80%, respectively. To our knowledge, this is the first study to evaluate the potential utility and clinical significance of CMI in the identification of MAFLD.

MAFLD was a redefinition of NAFLD in 2020 (1). This aroused great interest among scholars, and more evidence suggests that MAFLD was more than just a name change (33). In fact, the diagnosis of MAFLD included the concept of metabolic dysfunction in the human body. Our study found that the metabolism-related indicators including serum ALT, AST, TC, TG, HDL-C, LDL-C, FBG, SUA, and CMI of subjects in the MAFLD group were higher than those in the control group. The results were similar to previous studies (34–38). A study on the characteristics of MAFLD in adults over 40 years old in Shanghai, China found that metabolic disorders were more pronounced in the MAFLD group (39). The liver constitutes an essential organ in lipid metabolism. The hepatic steatosis is primarily caused by increased lipid acquisition and/or decreased lipid metabolism (40). Hepatic steatosis is a systemic metabolic disorder driven by adipocyte apoptosis. The lipids that cannot be metabolized by the liver will be transported to various organs of the body through the blood, and then lipids are deposited in these organs, leading to visceral obesity, adversely affecting multiple organs, and causing abnormalities in various metabolism-related indicators (41).

The CMI, as a new index to evaluate visceral obesity, was useful for assessing the risk of obesity-related metabolic diseases such as diabetes and CVDs (21, 26–28, 42). As previously mentioned, CMI was developed based on TG/HDL-C and WHtR that could easily be acquired. WHtR, as an abdominal obesity measurement index, was strongly associated with lipid content and lipid distribution and was superior to WC and BMI in the identification of NAFLD (24, 43). Additionally, previous studies confirmed that the TG/HDL-C was closely related to insulin resistance, obesity, and metabolic disorders and had a good predictive value for NAFLD (23). In addition, spearman correlation analysis showed that CMI levels were significantly correlated with MAFLD-related metabolic indicators and liver fat content detected by MRI, which further confirmed the close relationship between CMI and the risk of MAFLD.

Interestingly, further subgroup analyses demonstrated that there were significant additive interactions between CMI and MAFLD risk in gender, age, and BMI. The stronger correlations were found in female, young and nonobese participants. The ROC analyses also showed that there was a significantly better ability of CMI for identifying MAFLD among female, young and nonobese subjects. Similar to our findings, a cross-sectional study in the Pearl River Delta region of southern China found that CMI was a recommended indicator for screening women for nonalcoholic fatty liver disease (NAFLD) and could be used to detect high-risk NAFLD (44). As the metabolism, body composition, and coexisting diseases changed with aging (45–47), and the higher excessive fat accumulation of young people caused by dietary irregularities and insufficient exercise, it was reasonable that the performance of CMI may be influenced by age (48). As for the gender differences, one possible explanation may be related to the free fatty acids accumulation and metabolism, as females tend to have a greater contribution of visceral lipolysis to hepatic non-esterified fatty acid delivery to visceral fat than males (49). Additionally, different-sex hormones might affect the fat distribution which subsequently affects the association between CMI and MAFLD (50). At the same time, it was worth noting that subgroup analysis suggested that the WC, BMI, TG/HDL-C, and TG had similar results in the predictive ability of MAFLD in different genders. Moreover, the optimal cutoff values of these metabolic risk factor indicators and CMI for predicting MAFLD in females were all lower than those in males, which indirectly indicated that there were gender differences in the impact of metabolic disorders on MAFLD, and also broadened new ideas for the prevention and screening of MAFLD in different genders.

Regarding the differences in the predictive power of CMI for MAFLD among different obese subgroups, MAFLD is well known as an obesity-related disease and dose-dependently associated with visceral obesity (51), but it is worth noting that 6-20% of patients with MAFLD in clinical practice are neither overweight nor obese (2, 52). Previous studies also showed that lean people with unhealthy metabolism might have a greater accumulation of visceral fat (53), and nonobese MAFLD patients with unhealthy metabolism usually exhibited higher liver damage and cardiovascular risks (21). Thus, the CMI, as a more sensitive indicator than WC and BMI in reflecting the accumulation of visceral fat, the reason why it behaved a stronger association with nonobese MAFLD patients and had a better predictive ability in nonobese subjects may be explained. In addition, we also compared the predictive power of CMI with WC, BMI, TG/HDL-C, and TG in different subgroups. Interestingly, among nonobese subjects, the predictive power of CMI was significantly higher than that of WC, BMI, TG/HDL-C, and TG, which further confirmed and supported the excellent predictive value of CMI for nonobese MAFLD patients.

The **Supplementary Table 1** of this study illustrated that the optimal cutoff values of CMI for predicting MAFLD in different gender and age subgroups were different. However, for the convenience of clinical application and promotion, this study defined high CMI according to the optimal cutoff values for different genders, which were 0.6085 and 0.4319 for males and females, respectively. High CMI was defined when the CMI level was above the optimal cutoff values. At the same time, in order to avoid the variability caused by ultrasound examination, we also conducted further validation with MRI diagnosis in 131 subjects. The results showed that the accuracy, sensitivity, and specificity of high CMI for the diagnosis of MAFLD were 85.5%, 87.5%, and 80%, which further confirmed the excellent diagnostic performance of high CMI for MAFLD.

In summary, this study comprehensively evaluated the relationship between CMI and MALFD and confirmed that CMI had a superior predictive value for MAFLD, especially in females, young and nonobese people. Additionally, we also determined the best cutoff values of CMI for predicting MAFLD in both genders, which behaved excellent diagnostic performance for MALFD. CMI is composed of conventional blood lipid indexes and anthropometric indexes and has many advantages such as easy to obtain, low cost and high diagnostic accuracy. CMI is of great clinical value in early identification of MAFLD and is worthy of clinical application.

## Limitation

However, there were still some limitations in our study. First, this study was a cross-sectional study that cannot prove a causal relationship between CMI and MAFLD. At the same time, in order to avoid recall bias, this study mainly focused on the analysis of objective measurement indicators and biochemical indicators, and did not investigate the subjects’ diet, exercise and other situations with subjective recall. Second, this study used ultrasound as the diagnostic criteria for fatty liver in the training set, which could not accurately assess the severity of MAFLD, and could not further evaluate the relationship between CMI and the severity of MAFLD. Third, due to limited funding, the sample size of the validation set with MRI diagnosis was relatively small. Besides, the subjects included in this study were from a single center, and the age and weight of the included population might not be representative of all Chinese populations. Therefore, large multicenter prospective cohort studies are needed to explore the predictive value of CMI in different age and weight populations. In addition, further validation and exploration of the predictive value of CMI for MAFLD and its different severities are required in the future.

## Conclusion

CMI was strongly and positively associated with the risk of MAFLD and can be a reference predictor for MAFLD. High CMI had excellent diagnostic performance for MALFD, which can enable important clinical value for early identification and screening of MAFLD.

## Data availability statement

The data sets used and/or analyzed during the current study are available from the corresponding author on reasonable request.

## Ethics statement

This study has been approved by the Clinical Research Ethics Committee of China-Japan Friendship Hospital (2018-110-K79-1). The patients/participants provided their written informed consent to participate in this study.

## Author contributions

SD and DY contributed to the statistical analysis and wrote the manuscript. HX, JC, and ZR participated in the acquisition, analysis, or interpretation of data. SD and SY reviewed and edited the manuscript. SY is the guarantor of the work. SD and DY were the major contributors to finishing the manuscript. All authors read and approved the final manuscript.

## Conflict of interest

The authors declare that the research was conducted in the absence of any commercial or financial relationships that could be construed as a potential conflict of interest.

## Publisher’s note

All claims expressed in this article are solely those of the authors and do not necessarily represent those of their affiliated organizations, or those of the publisher, the editors and the reviewers. Any product that may be evaluated in this article, or claim that may be made by its manufacturer, is not guaranteed or endorsed by the publisher.

## Supplementary material

Supplementary Table 1 Analysis of the ROC curve results of CMI, WC, BMI, TG/HDL-C, and TG.

Supplementary Table 2 Correlation between CMI and MAFLD related indicators.
