# Supplementary material for: Cardiometabolic index: A new predictor for metabolic associated fatty liver disease in Chinese adults
Source: Front Endocrinol (Lausanne). 2022 Sep 16;13:1004855. doi: 10.3389/fendo.2022.1004855 (PMC9523727; doi:10.3389/fendo.2022.1004855)
Supplement: Supplementary Table 1 — Analysis of the ROC curve results of CMI, WC, BMI, TG/HDL-C, and TG. [file Table_1.docx]

Supplementary Material

**Supplementary Table 1** **|** Analysis of the ROC curve results of CMI, WC, BMI, TG/HDL-C, and TG.

| Variable | AUC (95%CI) | *P*-value | Sensitivity (%) | Specificity (%) | Youden index | Cutoff value |
| --- | --- | --- | --- | --- | --- | --- |
| Total |  |  |  |  |  |  |
| CMI | 0.802 (0.774 - 0.828) ^c d^ | <0.0001 | 75.00 | 73.86 | 0.4886 | 0.6112 |
| WC (cm) | 0.790 (0.761 - 0.817) | <0.0001 | 85.94 | 58.52 | 0.4446 | 88.00 |
| BMI (kg/m^2^) | 0.788 (0.759 - 0.814) | <0.0001 | 83.20 | 59.09 | 0.4229 | 25.10 |
| TG/HDL-C | 0.780 (0.751 - 0.807) | <0.0001 | 75.20 | 69.89 | 0.4508 | 1.0815 |
| TG | 0.773 (0.744 - 0.801) | <0.0001 | 67.38 | 74.72 | 0.4210 | 1.46 |
| Male |  |  |  |  |  |  |
| CMI | 0.774 (0.739 - 0.806) ^c d^ | <0.0001 | 79.44 | 63.91 | 0.4335 | 0.6085 |
| WC (cm) | 0.770 (0.735 - 0.803) | <0.0001 | 72.59 | 67.39 | 0.3998 | 92.50 |
| BMI (kg/m^2^) | 0.773 (0.738 - 0.805) | <0.0001 | 72.34 | 68.70 | 0.4103 | 26.32 |
| TG/HDL-C | 0.755 (0.719 - 0.788) ^d^ | <0.0001 | 75.89 | 64.78 | 0.4067 | 1.2080 |
| TG | 0.739 (0.702 - 0.773) | <0.0001 | 72.34 | 65.65 | 0.3799 | 1.45 |
| Female |  |  |  |  |  |  |
| CMI | 0.853 (0.801 - 0.895) ^c^ | <0.0001 | 79.66 | 80.33 | 0.5999 | 0.4319 |
| WC (cm) | 0.820 (0.765 - 0.866) | <0.0001 | 81.36 | 69.67 | 0.5103 | 85.50 |
| BMI (kg/m^2^) | 0.804 (0.748 - 0.852) | <0.0001 | 88.98 | 58.20 | 0.4718 | 23.34 |
| TG/HDL-C | 0.819 (0.764 - 0.865) | <0.0001 | 82.2 | 70.49 | 0.5270 | 0.7261 |
| TG | 0.829 (0.775 - 0.874) | <0.0001 | 72.03 | 81.15 | 0.5318 | 1.15 |
| Age≥45 y |  |  |  |  |  |  |
| CMI | 0.724 (0.666 - 0.777) ^c d^ | <0.0001 | 77.42 | 63.29 | 0.4071 | 0.5720 |
| WC (cm) | 0.694 (0.635 - 0.749) | <0.0001 | 51.61 | 77.22 | 0.2883 | 95.50 |
| BMI (kg/m^2^) | 0.716 (0.658 - 0.770) | <0.0001 | 70.97 | 64.56 | 0.3552 | 26.03 |
| TG/HDL-C | 0.701 (0.642 - 0.755) | <0.0001 | 72.04 | 64.56 | 0.3660 | 1.0882 |
| TG | 0.689 (0.629 - 0.744) | <0.0001 | 66.13 | 65.82 | 0.3195 | 1.50 |
| Age<45 y |  |  |  |  |  |  |
| CMI | 0.827 (0.794 - 0.856) ^c d^ | <0.0001 | 73.93 | 78.02 | 0.5195 | 0.6367 |
| WC (cm) | 0.821 (0.788 - 0.851) | <0.0001 | 80.06 | 69.96 | 0.5002 | 90.00 |
| BMI (kg/m^2^) | 0.811 (0.777 - 0.842) | <0.0001 | 81.9 | 66.67 | 0.4857 | 25.39 |
| TG/HDL-C | 0.807 (0.773 - 0.838) | <0.0001 | 76.99 | 71.79 | 0.4879 | 1.0815 |
| TG | 0.797 (0.762 - 0.828) | <0.0001 | 70.55 | 75.46 | 0.4601 | 1.38 |
| BMI≥28 kg/m^2^ |  |  |  |  |  |  |
| CMI | 0.593 (0.533 - 0.651) ^c^ | 0.0439 | 38.24 | 79.55 | 0.1778 | 1.2142 |
| WC (cm) | 0.597 (0.688 - 0.780) | 0.0303 | 36.97 | 79.55 | 0.1652 | 104.50 |
| BMI (kg/m^2^) | 0.648 (0.589 - 0.704) | 0.0005 | 65.13 | 68.18 | 0.3331 | 29.38 |
| TG/HDL-C | 0.579 (0.519 - 0.638) | 0.0849 | 25.63 | 90.91 | 0.1654 | 2.6000 |
| TG | 0.590 (0.530 - 0.648) | 0.0573 | 53.36 | 65.91 | 0.1927 | 1.82 |
| BM<28 kg/m^2^ |  |  |  |  |  |  |
| CMI | 0.801 (0.767 - 0.833) ^abcd^ | <0.0001 | 75.91 | 73.38 | 0.4929 | 0.5304 |
| WC (cm) | 0.754 (0.717 - 0.789) | <0.0001 | 78.10 | 63.31 | 0.4141 | 87.50 |
| BMI (kg/m^2^) | 0.733 (0.695 - 0.768) ^c^ | <0.0001 | 77.37 | 59.42 | 0.3679 | 24.56 |
| TG/HDL-C | 0.783 (0.747 - 0.816) | <0.0001 | 69.71 | 76.30 | 0.4601 | 1.0815 |
| TG | 0.778 (0.742 - 0.811) | <0.0001 | 66.79 | 76.95 | 0.4374 | 1.38 |

CMI: cardiometabolic index; WC: waist circumference; BMI: body mass index; TG: triglyceride; HDL-C: high-density lipoprotein cholesterol.

^a^ indicates significantly larger compared with WC.

^b^ indicates significantly larger as compared with BMI.

^c^ indicates significantly larger as compared with TG/HDL-C.

^d^ indicates significantly larger as compared with TG.
