# Supplementary material for: Cardiometabolic index: A new predictor for metabolic associated fatty liver disease in Chinese adults
Source: Front Endocrinol (Lausanne). 2022 Sep 16;13:1004855. doi: 10.3389/fendo.2022.1004855 (PMC9523727; doi:10.3389/fendo.2022.1004855)
Supplement: Supplementary Table 2 — Correlation between CMI and MAFLD related indicators. [file Table_2.docx]

Supplementary Material

**Supplementary Table 2 |** Correlation between CMI and MAFLD related indicators (r value)

| Variable | CMI | *P*-value |
| --- | --- | --- |
| WC(cm) | 0.575 | <0.01 |
| BMI(kg/m^2^) | 0.555 | <0.01 |
| SBP (mmHg) | 0.321 | <0.01 |
| DBP (mmHg) | 0.340 | <0.01 |
| TC (mmol/L) | 0.189 | <0.01 |
| TG (mmol/L) | 0.946 | <0.01 |
| HDL-C (mmol/L) | -0.612 | <0.01 |
| LDL-C (mmol/L) | 0.247 | <0.01 |
| FBG (mmol/L) | 0.316 | <0.01 |
| SUA (μmol/L) | 0.462 | <0.01 |
| ALT (U/L) | 0.488 | <0.01 |
| AST (U/L) | 0.322 | <0.01 |

CMI: cardiometabolic index; WC: waist circumference; BMI: body mass index; SBP: Systolic blood pressure; DBP: diastolic blood pressure; TC: total cholesterol; TG: triglyceride; HDL-C: high-density lipoprotein cholesterol; LDL-C: low-density lipoprotein cholesterol; FBG: fasting blood glucose; SUA: serum uric acid; ALT: alanine aminotransferase; AST: aspartate aminotransferase.
